# Evaluation of radiation dose to patients undergoing interventional radiology procedures at Ramathibodi Hospital, Thailand

**DOI:** 10.2349/biij.7.3.e22

**Published:** 2011-07-01

**Authors:** J Urairat, S Asavaphatiboon, S Singhara Na Ayuthaya, N Pongnapang

**Affiliations:** 1 School of Medical Physics, Faculty of Medicine Ramathibodi Hospital, Mahidol University, Bangkok, Thailand; 2 Department of Radiology, Faculty of Medicine, Ramathibodi Hospital, Mahidol University, Bangkok, Thailand; 3 Department of Radiological Technology, Faculty of Medical Technology, Mahidol University, Bangkok, Thailand

**Keywords:** Radiation doses, Patient doses, Air Kerma Area Product (KAP), Entrance dose, Interventional Radiology

## Abstract

**Purpose:**

This study was carried out to assess the radiation dose to patients undergoing interventional radiology procedures at Ramathibodi Hospital, Bangkok, Thailand.

**Methods:**

Data were collected from 60 patients under transarterial oily-chemoembolisation (TOCE) and femoral angiography performed with the Toshiba Infinix model VC-i FPD single plane system. Data were also collected from 60 patients who underwent brain arteriovenous malformations (AVM) and dural-arteriovenous fistula (DAVF) embolisation, performed with the Toshiba Infinix model VF-i bi-plane systems. A built-in air kerma area product (KAP) meter calibrated *in situ* was used for the skin dose calculation.

**Results:**

The calibration coefficient of air kerma area product meter at tube voltage between 50 kV and 100 kV was found to vary within ± 5.07%, ± 7.2%, ± 4.86 % from calibration coefficient of 80 kV for a single-plane, tube 1 and tube 2 of bi-plane x-ray system, respectively. Mean air kerma area product values were 90.99 ± 52.89, 31.02 ± 17.92, 33.11 ± 23.99 (Frontal), 35.01 ± 19.10 (Lateral), 50.15 ± 44.76 (Frontal), 97.31 ± 44.12 (Lateral) Gy-cm^2^ for transarterial oily-chemoembolisation, femoral angiography, diagnostic cerebral angiography, therapeutic cerebral angiography, respectively. The therapeutic cerebral angiography procedure was found to give the highest entrance dose, number of images and fluoroscopy time: 362.63 cGy (Lateral), 1015 images (Lateral) and 126 minutes, respectively. However, the highest air kerma area product value was from transarterial oily-chemoembolisation with 264.37 Gy-cm^2^. There were 2 cases of therapeutic cerebral angiography, where the patient entrance dose was higher than 3 Gy in the frontal view, which reached the deterministic threshold for temporary epilation.

**Conclusion:**

Very wide variationswere found in patient dose from different interventional procedures. There is a need for a dose record system to provide feedback to radiologists who perform the procedures; especially in cases where the dose exceeds the deterministic threshold.

## INTRODUCTION

In 2006, the National Council on Radiation Protection and Measurements (NCRP) published a report titled [1] ‘Ionizing Radiation Exposure of the Population of the United States’. The report showed that human-made radiation contributes 50% of the radiation in our environment. Of this human-made radiation, 36% comes from diagnostic radiation, including 5% from general X-rays and fluoroscopy, 24% from computed tomography (CT), and 7% from interventional radiology. It is clear that the biggest amount of radiation received by the population comes from general diagnostic radiation. Nonetheless, interventional radiology, though contributing a lesser extent of radiation delivery to the population, can emit very high individual doses that can possibly reach the deterministic threshold.

Currently, interventional radiology procedures are increasingly performed in a number of pathological conditions. However, the high radiation dose from lengthy procedures can harm patients. Physicians are concerned about serious radiation-induced injury caused by high radiation skin dose or lengthy exposure to fluoroscopy in some procedures. In light of such concerns, the World Health Organization (WHO) and the German Institute for Radiation Hygiene (ISH) [2] organised a workshop on ensuring efficacy and radiation safety in interventional radiology. The Food and Drug Administration (FDA) published documents [3] on avoiding deterministic effects in cardiology procedures. The absorbed dose in the patient’s skin of 2 Gray (Gy) should raise concerns about the onset of transient erythema and 3 Gy for temporary epilation. [3] Information about the absorbed dose to skin should be included in the patient’s records.

A number of investigators have utilised the air kerma area product values to assess radiation risk from fluoroscopic studies [4–9]. The air kerma area product is a better indicator of risk, compared to the entrance skin dose, due to the fact that air kerma area product is a product of entrance skin dose and field size [10]. This study was carried out to assess the radiation dose to patients undergoing interventional radiology procedures at Ramathibodi Hospital, Bangkok, Thailand.

## MATERIALS AND METHODS

### X-ray system

The evaluation of patient dose was carried out at the radiology department of Ramathibodi Hospital. The interventional procedures were performed using two radiographic systems. The transarterial oily-chemoembolisation and femoral angiography procedures were performed using the Toshiba Infinix VC-I FPD with X-ray generator model XTP 8100G (single-plane x-ray system), whereas diagnostic cerebral angiography and therapeutic cerebral angiography (brain arteriovenous malformations and duralarteriovenous fistula embolization) were performed using Toshiba Infinix VB-I with X-ray generator model XTP 8100G (bi-plane x-ray system). The exposure parameters, such as tube potential and tube current, were controlled by automatic brightness control (ABC). The radiographic systems utilised 4 field diameters including 6 inches, 8 inches, 12 inches, and 16 inches for Infinix VC-I FPD; and 5 inches, 7 inches, 9 inches and 12 inches for Infinix VB-I. All X-ray tubes were equipped with built-in air kerma area product meter (Diamentor K1S) at the collimator exit with capability to display the cumulative air kerma area product for fluoroscopic and radiographic examinations. The half-value-layer (HLV), tested at tube potential of 80 kVp and 74 kVp prior to the air kerma area product calibration, were 4.0 mmAl and 3.7 mmAl for Infinix VC-I FPD and Infinix VB-I, respectively.

### Air Kerma Area Product meter calibration

Built-in air kerma area product meter was calibrated *in situ* with a calibrated ionisation chamber (Accu-Pro^TM^). In this calibration procedure, a Fuji image photo-stimulable phosphor plate was exposed and read by a Fuji FCR 5000 pulse for beam area determination. The calibration procedures and beam area determination were recommended by the International Atomic Energy Agency (IAEA TRS 457) [10]. The calibration coefficient of air kerma area product meter (N_KAP_) at tube voltage between 50 kV and 100 kV was found to vary within ± 5.07%, ± 7.20%, ± 4.86% from calibration coefficient of 80 kV for Infinix VC-I FPD, Infinix VB-I tube 1 and tube 2 respectively.

### Patient data collection

A total of 120 patients (78 male, 42 female, mean age of 51.29 ± 16.88 and 53.43 ± 20.94 years for male and female, respectively) who underwent interventional radiology procedures participated in this study and signed a consent form. Ethical clearance was obtained from the Ramathibodi Hospital research ethics committee. Patient-specific data, i.e., couch position, field of view (FOV), kerma area product, and tube potential were collected from the displayed monitor for purpose of dose calculation.

Air kerma area product was corrected with calibration coefficient of air kerma area product meter for average tube potential of each projection. Entrance skin dose (ESD) was calculated from air kerma area product (${\text{M}}_{\text{Q}}^{\text{KAF}}$ ), beam area (A_nam_) and calibration coefficient of air kerma area product meter (${\text{N}}_{{\text{P}}_{\text{XA}}\text{Q}}$ ) at calibrated distance (see equation of entrance dose below) to focal-to-skin distance (FSD) by inverse square law and multiplied by backscatter factor for each series. Backscatter factor was interpolated from the tabulated data by using half value layer, and the focal-to-skin distance was calculated from the couch position.

$$ED=\frac{M_{\text{Q}}^{\text{KAP}}N_{{\text{P}}_{\text{XA}}\text{Q}}}{A_{nam}}$$

## RESULTS AND DISCUSSION

Table 1 shows the mean, median and standard deviation values of the kVp, mA, ms, and field of view (FOV). The mean values of tube potential for transarterial oily-chemoembolisation, femoral angiography, diagnostic cerebral angiography, therapeutic cerebral angiography are 82.9, 80.3, 76 (frontal), 74 (lateral), 75.6 (frontal) and 74.6 (lateral) kVp, respectively. The mean, median,****maximum and standard deviation values of air kerma area product (KAP), entrance skin dose (ESD), number of images and fluoroscopy time are shown in Table 2 and 3.

**Table 1:** The mean, the median and the standard deviation values of the kVp, mA, ms, Field of View (FOV).

	**kVp**			**mA**			**ms**			**FOV**		
	**mean**	**median**	**SD**	**mean**	**median**	**SD**	**mean**	**median**	**SD**	**mean**	**median**	**SD**
TOCE	82.9	82	5.03	124.2	125	25.67	69.1	74.9	18.43	13.4	12	2.59
Femoral angiography	80.3	80	1.91	79.9	80	43.55	21.8	10	21.99	15.9	16	0.49
Diagnostic cerebral angiography												
Frontal	76	74	3.75	259.3	160	156.44	54.7	63	23.32	9	9	1.21
Lateral	74	74	0.00	399.4	400	7.18	41.3	40	10.65	10	9	1.59
Therapeutic cerebral angiography												
Frontal	75.6	74	2.57	217.3	160	110.48	63.6	66	16.44	7.7	9	2.06
Lateral	74.6	74	2.38	380.8	400	70.55	46.3	43	18.23	8.7	9	2.58

**Table 2:** The mean, the median and the standard deviation values of air kerma-area product (KAP), Entrance dose (ED), Number of images and Fluoroscopy time.

	**No.**	**KAP (cGy-cm^2^)**			**ED (mGy)**			**Number of images**			**Fluoroscopy time (min)**		
		**mean**	**median**	**SD**	**mean**	**median**	**SD**	**mean**	**median**	**SD**	**mean**	**median**	**SD**
TOCE	30	9099.41	7719.30	5288.79	394.86	339.55	292.28	104.83	97.5	45.71	14.94	8.8	12.87
Femoral angiography	30	3101.87	2355.93	1791.89	100.36	78.21	59.65	220.73	195.5	71.00	5.9	4.95	4.11
Diagnostic cerebral angiography													
Frontal	30	3310.97	2724.04	2398.89	400.40	308.65	318.48	378.23	284.5	287.26	13.27	12.25	7.25
Lateral		3500.68	3328.83	1909.86	154.77	125.96	93.56	118.13	110.5	37.24			
Therapeutic cerebral angiography													
Frontal	30	5014.75	3706.52	4476.37	876.52	718.48	773.43	354.06	295.5	224.42	40.1	32.8	27.84
Lateral		9731.47	9404.30	4411.78	591.93	606.57	312.42	334.73	317.5	177.97			

*n = 33, percentages in parentheses.

**Table 3:** Mean Fluoroscopy, KAP and ED of this study and other study.

**Procedure**		**Study**	**Mean Fluoroscopy time (min)**	**Mean KAP (Gy-cm^2^)**	**Mean ED (mGy)**
TOCE		This study	14.94	90.99	394.86
		Wongsanon S., et al. [12]	8.58	121.09	370
		Donald L., et al.[13]	16.8	282.32	1,406
		Vano et al. [14]	-	81.7	500
Femoral angiography		This study	5.9	31.02	100.36
		Vano et al. [14]	-	66.6	-
		Ioaninis A. Tsalafoutas, et al. [15]	2.6	68	257
		McParland [16]	7.2	46.7	-
Diagnostic cerebral angiography	Frontal	This study	13.27	33.11	400.40
	Lateral			35.01	154.77
		Ioaninis A. Tsalafoutas, et al. [15]	4.3	50	349
		McParland [16]	12.1	74.1	220
		Marshall, et al. [16]	-	48.5	150
Therapeutic cerebral angiography	Frontal	This study	40.1	50.15	876.52
	Lateral			97.31	591.93
		Donald L., et al.[13]	92.5	339.76	3791
		McParland [16]	34.1	105	-
		Marshall, et al. [16]	-	122	-

In this study, the highest entrance dose, number of images and fluoroscopy time of 362.63 cGy, 1015 images and 126 minutes, respectively, were found in the lateral tube position of therapeutic cerebral angiography. However, the highest air kerma area product was from transarterial oily-chemoembolisation with a value of 264.37 Gy-cm^2^. There were two patients who received entrance doses higher than 3 Gy in a frontal view of therapeutic cerebral angiography. This dose level is higher than a threshold dose for temporary epilation.

From the results, wide variations (9.89–230.00 Gy-cm^2^ and 15.47–362.63 cGy in therapeutic cerebral angiography) were found in air kerma area product and entrance skin dose. Complicated procedures require more operating time and deliver higher radiation doses to patients, compared to common and non-complicated procedures. Differences in the irradiated field size can also cause the difference in radiation dose to patients. In this study, it was found that larger irradiated fields such as transarterial oily-chemoembolisation produced higher air kerma area product than the smaller field in therapeutic cerebral angiography. However, the entrance skin dose from transarterial oily-chemoembolisation was smaller, as it required less irradiation time. Table 4 shows the comparison between air kerma area product and entrance skin dose from this study with other published data. The mean air kerma area product of this study was lower than other studies in transarterial oily-chemoembolisation and femoral angiography except when compared with Veno’s study in transarterial oily-chemoembolisation. In diagnostic and therapeutic cerebral angiography procedures, the mean air kerma area product was lower than in other studies, when compared with each tube. But it was higher than that of some studies when compared with both tubes.

To utilise the displayed air kerma area product values from the console screen, with proper calibration values, conversion of air kerma area product to entrance skin dose can be carried out and used. In this study, to set up a concerned level for deterministic risk of 2 Gy, correlations (R^2^) between the air kerma area product values and the calculated entrance skin dose of 0.83, 0.98, 0.86, 0.77, 0.92, 0.69 for transarterial oily-chemoembolisation, femoral angiography, diagnostic cerebral angiography frontal and lateral, therapeutic cerebral angiography frontal and lateral, respectively, were found. The corresponding air kerma area product values were 410.77, 606.66, 162.73, 464.11, 118.12 and 335.21 Gy-cm^2^.

## CONCLUSION

High-dose radiological procedures such as interventional radiology have been known to expose patients to high doses of radiation. The high dose levels can sometimes reach the deterministic risk threshold. Air kerma area product meters are now commonly incorporated with the fluoroscopic unit to provide feedback on radiation dose emitted from the x-rays tube. The air kerma area product meter calibrated using proper *in situ* protocol can be used as a monitoring tool to alert clinicians regarding deterministic risk of radiation such as early transient erythema or epilation.

## References

[1] National Council on Radiation Protection and Measurements NCRP Report No. 160 Pie Charts [Online]..

[2] (1997). Joint WHO/ISH Workshop on Efficacy and Radiation Safety in Interventional Radiology, 9-13 October 1995.

[3] US Food and Drug Administration (1994). Avoidance of serious X-ray-induced skin injuries to patients during fluoroscopically guided procedures. Med Bull.

[4] Crawley MT, Mutch S, Nyekiova M, Reddy C, Weatherburn H (2001). Calibration frequency of dose–area product meters. Brit J Radiol.

[5] Vano E, Sanchez R, Fernandez JM, Gallego JJ, Verdu JF, de Garay MG, Azpiazu A, Segarra A, Hernandez MT, Canis M, Diaz F, Moreno F, Palmero J (2009). Patient dose reference levels for interventional radiology: A national approach. Cardiovasc Intervent Radiol.

[6] Bor D, Sancak T, Olgar T, Elcim Y, Adanali A, Sanlidilek U, Akyar S (2004). Comparison of effective doses obtained from dose-area product and air kerma measurements in interventional radiology. Brit J Radiol.

[7] van de Putte S, Verhaegen F, Taeymans Y, Thierens H (2000). Correlation of patient skin dose in cardiac interventional radiology with dose-area product. Brit J Radiol.

[8] Bor D, Olğar T, Toklu T, Cağlan A, Onal E, Padovani R (2009). Patient doses and dosimetric evaluations in interventional cardiology. Physica Medica.

[9] Ruiz Cruces R, García-Granados J, Diaz Romero FJ, Hernández Armas J (1998). Estimation of effective dose in some digital angiographic and interventional procedures. Brit J Radiol.

[10] International Atomic Energy Agency (2007). Dosimetry in diagnostic radiology: an international code of practice.

[11] Wongsanon S, Witchathorntakun W, Wongsanon W (2009). Patient radiation dose received from the body interventional radiology. Srinagarind Med J.

[12] Miller DL, Balter S, Cole PE, Lu HT, Schueler BA, Geisinger M, Berenstein A, Albert R, Georgia JD, Noonan PT, Cardella JF, St George J, Russell EJ, Malisch TW, Vogelzang RL, Miller GL, Anderson J (2003). Radiation doses in interventional radiology procedures: The RAD-IR study: Part I: Overall measures of dose. J Vasc Interv Radiol.

[13] Vañó E, González L, Fernández JM, Guibelalde E (1995). Patient values in interventional radiology. Brit J Radiol.

[14] Tsalafoutas IA, Goni H, Maniatis PN, Pappas P, Bouzas N, Tzortzis G (2006). Patient Doses from Non-cardiac Diagnostic and Therapeutic Interventional Procudures. J Vasc Interv Radiol.

[15] Mcparland BJ (1998). A study of patient radiation doses in interventional radiological procedures. Brit J Radiol.

[16] Marshall NW, Noble J, Faulkner K (1995). Patient and staff dosimetry in neuroradiological procedures. Brit J Radiol.

